# Administrative process reform in eastern coastal Chinese public hospitals: a policy and practice review

**DOI:** 10.3389/fpubh.2026.1810349

**Published:** 2026-05-25

**Authors:** Guangchao Ding, Yijing Ling, Owusu Mensah Solomon

**Affiliations:** 1 The Second Affiliated Hospital of Zhejiang Chinese Medical University, Hangzhou, China; 2 Nursing College, Zhejiang Chinese Medical University, Hangzhou, China; 3 International Education College, Zhejiang Chinese Medical University, Hangzhou, China

**Keywords:** administrative management, administrative MDT, high-quality development, large-department system reform, process optimization, public hospitals

## Abstract

China's public hospitals are currently at a crucial point in their journey toward high-quality development. The operational efficiency of administrative processes directly impacts overall hospital performance and healthcare service quality. Constrained by traditional hierarchical management models, some public hospitals still face practical challenges such as lengthy approval procedures, difficulties in interdepartmental coordination, barriers to information sharing, and misaligned service awareness. The lengthiness and inefficiency of administrative processes not only make it difficult to provide clinical services but also encroach on healthcare professionals' time for patient care, increasing their non-clinical workload. This, in turn, affects the patient experience and patient safety and has become a major obstacle to the high-quality development of hospitals. However, existing research has largely focused on optimizing individual functional modules or the application of specific management tools, lacking in-depth exploration of holistic and systematic improvements to administrative processes. Furthermore, there is a scarcity of integrative research that combines international best practices with China's local bureaucratic culture, personnel systems, and IT infrastructure, resulting in a lack of practical guidance and replicability. This study focuses on administrative process reform in public hospitals, employing a case study approach. It examines the administrative practices of a large public hospital in a coastal city in eastern China as its research case. It systematically examines the hospital's specific operational plans, innovative practices, and practical outcomes in establishing an administrative Multidisciplinary Diagnosis and Treatment Team (MDT) collaborative governance mechanism, reengineering multi-functional service processes, and restructuring logistics departments under a large-department system. Concurrently, it compares international literature to analyze administrative reform experiences from advanced healthcare management nations like Singapore and Japan, conducting localized qualitative analysis for adaptation. At the theoretical level, this study employs a descriptive single-case design based on secondary data to expand the application and interpretation of bureaucratic theory, collaborative governance theory, and organizational process reengineering theory in the administrative context of public hospitals. At the practical level, unlike existing research that tends to focus on policy recommendations, this study systematically presents a comprehensive operational framework for administrative MDTs—covering institutional design, operational processes, and performance evaluation and oversight. It reveals the interplay between process reengineering and digital transformation, as well as between technological empowerment and institutional restructuring, and has tentatively established a reform pathway that combines an international perspective with local adaptability which may enhance the efficiency and effectiveness of public hospital administration. Building on this foundation, it proposes a public hospital administrative process reform strategy centered on system restructuring, technological empowerment, and talent cultivation. It also outlines the core principles that reforms must adhere to, with the aim of providing a preliminary practical reference for public hospitals with similar conditions to break free from the constraints of traditional management thinking and establish a modern administrative management system that is responsive, flexible, and efficient.

## 1 Introduction

China's 15th Five-Year Plan proposal advocates “implementing a health-first development strategy.” The State Council General Office's Opinions on Promoting High-Quality Development of Public Hospitals [Guobanfa (2021) No. 18] explicitly requires public hospitals to transition from scale expansion to quality enhancement and efficiency improvement, and from extensive management to refined management (1). The 15th Five-Year Plan period focuses on building a Healthy China, striving to address prominent shortcomings such as weak primary healthcare services and regional development imbalances by implementing targeted policies and investments aimed at strengthening healthcare infrastructure and improving access to medical services across all regions. Against this backdrop, public hospitals—as the backbone of China's healthcare service system—play a pivotal role. Their administrative management efficiency directly determines the quality of medical services and operational effectiveness, serving as a crucial safeguard for achieving high-quality development.

However, due to inherent flaws in the traditional hierarchical management model, some public hospitals still suffer from lengthy processes, poor interdepartmental coordination, and high management costs within their internal administrative systems. These issues not only reduce administrative efficiency but also indirectly impact the service experience of clinical diagnosis and treatment (1, 2). Weber's theory of bureaucracy (3) highlights the advantages of characteristics such as hierarchical control and clear rules in enhancing organizational efficiency, but it also points out that this system is prone to rigidity and departmental fragmentation when faced with demands for complexity and flexibility. Given the higher demands placed on organizational responsiveness and collaborative capabilities by the current push for high-quality development in public hospitals, relying solely on functional adjustments within the bureaucratic framework is no longer sufficient to overcome institutional bottlenecks. Current industry research on hospital administration primarily focuses on optimizing individual functional modules or applying specific management tools, with relatively limited exploration of holistic, systematic improvements to administrative processes. There is a lack of in-depth analysis regarding concrete implementation pathways, operational steps, and supporting mechanisms for reform execution. Furthermore, there has been a scarcity of integrative research that combines international best practices and theories with China's unique bureaucratic culture, personnel systems, and IT infrastructure to facilitate localized operational implementation. Consequently, existing research findings lack sufficient practical guidance and replicability. Therefore, this study incorporates the theories of collaborative governance and organizational process reengineering (4, 5), treating administrative process reform as a systematic transformation process aimed at breaking down bureaucratic barriers and establishing horizontal collaboration mechanisms. It focuses on the institutional pathways for cross-departmental collaboration and the operational logic of process restructuring, thereby providing a clear theoretical perspective and analytical framework for the research.

Although existing research yields useful insights for optimizing public hospital management, significant research gaps remain. In terms of research perspectives, the current literature predominantly focuses on efficiency improvements within single functional domains such as healthcare quality management, human resource management, or supply chain management. There is a lack of holistic, cross-departmental process reengineering studies that treat administrative management as a complex adaptive system which is essential for understanding the interdependencies and interactions between different departments in public hospitals. Methodologically, most studies remain at the level of problem description and policy recommendations, lacking in-depth case analyses of how reforms are implemented—such as specific operational plans, implementation steps, resistance management, and supporting mechanisms. In policy discussions, while existing policy documents and academic research have addressed the issue of management efficiency in public hospitals, they still lack a systematic response to the core challenge of administrative process reform: namely, how to institutionalize and normalize cross-departmental collaboration within a hierarchical structure. Policy initiatives have largely focused on operational aspects such as functional integration and IT infrastructure development, while insufficient attention has been paid to deeper-rooted obstacles that may arise during the reform process—such as entrenched departmental interests, resistance to the restructuring of authority and responsibilities, and the need to shift staff mindsets. Furthermore, there has been a lack of critical examination of the mechanisms that ensure alignment between reform pathways and the institutional environment which is essential for addressing the deeper-rooted obstacles that hinder effective implementation of policy initiatives. Therefore, pressing questions that demand answers at both theoretical and practical levels include how to systematically resolve the coordination challenges in public hospital administrative processes and how to construct a reform pathway that combines international perspectives with local feasibility.

From an international perspective, countries with mature healthcare systems such as Singapore, Japan, and the United Kingdom have developed numerous valuable experiences in optimizing administrative processes within public hospitals: Singapore's public hospitals implement a “patient-centered” one-stop administrative service model, centralizing administrative tasks through cross-departmental service centers while leveraging a unified national medical data platform to break down information silos (6, 7); Japanese public hospitals emphasize deep integration between administrative and clinical functions, establishing a clinical rotation system for administrative staff and cross-departmental collaborative assessment mechanisms to institutionally reinforce service orientation toward clinical needs (8). The UK's National Health Service (NHS) integrates logistical and administrative functions through a “grand ministry” structure, building a centralized healthcare support system that significantly reduces management costs (9). Recently, the NHS has innovated service models in emergency and primary care, enhancing patient flow efficiency and resource allocation capabilities through standardized processes and big data support (10). The core logic of these international experiences largely revolves around “collaboration, digitalization, and service orientation,” aligning closely with the core demands of China's public hospital administrative reforms and providing important reference models for the country.

Based on this, this paper examines the administrative process reform practices at a public hospital in a coastal city in eastern China, identifies key issues in the current operation of administrative processes at public hospitals, It focuses on dissecting the hospital's specific operational plans, implementation steps, and supporting measures in core reform areas such as administrative multidisciplinary teams (MDTs), multi-functional position restructuring, and a centralized logistics department. By integrating and adapting international best practices to the local context, the paper summarizes the practical experiences and outcomes of these reforms. It then proposes preliminary reform pathways and strategies to provide a reference framework for administrative management reforms in public hospitals facing similar circumstances.

## 2 Methods

This study employs a descriptive case study approach based on secondary data, with all data drawn from publicly available literature, policy documents, and information released by hospitals. It aims to systematically analyze the practical pathways and operational mechanisms of administrative process reform in public hospitals.

### 2.1 Criteria for case selection

This study selected a large public hospital in a coastal city in eastern China as its research subject. The hospital is a Grade III Level A facility (the highest level in China's hospital grading system), referring to large teaching hospitals affiliated with institutions of higher education that typically provide treatment for complex and critical cases as well as undertake medical education and research. It has over 1,500 beds and an annual outpatient volume exceeding two million visits, playing a significant role in the local healthcare system. This selection was primarily based on the following considerations: First, the hospital in the case is a good example. As a Grade A, Level 3 public hospital serving a large population with a high volume of operations, the hospital concentrates issues such as lengthy approval processes, difficulties in interdepartmental coordination, and barriers to information sharing—problems that reflect the management challenges commonly faced by large public hospitals in China. Second, the case hospital's reforms are pioneering. Since 2021, the hospital has systematically advanced administrative process reforms, developing a series of concrete and actionable practices in areas such as establishing administrative MDT collaborative governance mechanisms, reengineering multi-functional service processes, and restructuring the logistics department into a large-scale organizational unit, thereby providing rich practical material for the study. Third, the information in this case study is readily accessible. The court consistently publishes updates on its reform efforts and performance data through various channels, including its official website, annual work reports, materials from academic conferences, and articles in academic journals. Research teams can obtain comprehensive and up-to-date information on the court's reforms through these public channels, which ensures the in-depth conduct of this case study.

### 2.2 Data collection methods

This study primarily employs a literature review approach. Data sources include: publicly available academic literature retrieved from databases such as China National Knowledge Infrastructure (CNKI), Wanfang Data, and Web of Science, covering journal articles and theses on topics such as administrative reform in public hospitals, the administrative MDT model, and the reform of the large-department system; policy documents and industry reports, including policy documents on public hospital reform issued by the State Council and relevant departments, as well as management guidelines and research reports published by professional associations, retrieved from government official websites and professional association websites; publicly available hospital information, including reform updates, annual work reports, and materials from academic conferences published on hospital websites, as well as relevant papers on administrative MDT reforms published in academic journals by research teams from the case hospitals.

### 2.3 Data analysis methods

This study employs a combined approach of content analysis and comparative analysis. Based on collected public literature and publicly available hospital information, it conducts an in-depth analysis of a single case to reveal the operational mechanisms of organizational change. Through comparison with international experiences, the study then identifies reform pathways with universal applicability. First, the data was organized and categorized. The collected academic literature, policy documents, and publicly available hospital information were sorted by source, type, and theme, and key information was extracted focusing on three core areas: the operational mechanisms of administrative MDTs, the process reengineering of multi-functional positions, and the reform of the centralized logistics department. The second step involves descriptive analysis. Based on the organized data, the study systematically examines the hospital's specific practices in the three major reform areas. Concurrently, by integrating statistical data reported in the literature, it identifies changes in areas such as approval efficiency and service experience before and after the reforms. The third step is comparative analysis. The case hospital's reform practices undergo a comparison with advanced experiences from countries like Singapore, Japan, and the United Kingdom. Differences and similarities are analyzed across dimensions including coordination mechanisms, process reengineering, and functional integration, clarifying the conditions for localizing international experiences and the methods for their adaptation. Step 4 involves comprehensive synthesis. Building on the descriptive and comparative analyses, this step distills the core strategies and fundamental principles of administrative process reform in public hospitals, thereby establishing a reform pathway with universal applicability.

### 2.4 Key issues in public hospital administrative processes

The operational contradictions in current public hospital administrative processes manifest not only as low efficiency at the execution level but more critically as systemic bottlenecks at the management level. While superficially evident in frontline staff facing difficulties and excessive legwork, the root cause lies in inadequate administrative effectiveness—a key factor constraining the high-quality development of China's public hospitals. Field research conducted at a large public hospital in an eastern coastal city reveals that these contradictions primarily manifest in four specific areas:

#### 2.4.1 Lengthy approval processes and low response efficiency

Frontline staff frequently report slow approval efficiency as the most pressing issue. Survey data (11, 12) indicates that 58.7% of employees believe internal approvals are excessive and urgently require streamlining and consolidation, while 51.7% report approval processes take too long to meet the real-time demands of clinical work. The core issue lies in the hierarchical management model, where traditional sequential approvals circulate through fixed levels across departments and between superiors and subordinates. This lacks horizontal coordination mechanisms and flexible contingency plans, which are essential for improving communication and responsiveness across departments in the approval process. Rigid process design has devolved into a mechanical “signature relay,” failing to achieve efficient approval control while causing administrative inefficiency, delaying progress, and hindering timely responses to frontline needs. For instance, when clinical departments apply for temporary equipment procurement, the process must sequentially pass through multiple approval stages—department heads, the Medical Affairs Department, the Equipment Department, and hospital leadership—resulting in lengthy average approval times that fall far short of meeting urgent clinical needs. This issue is prevalent across public hospitals in China. This finding is consistent with the conclusions of studies by Yanling and Yuanbo et al. (1, 11), all of which point to the problems of excessive hierarchy and slow response times in existing approval processes. However, this study further reveals that the root cause of approval inefficiency lies not in the speed of individual nodes, but in the design of the serial workflow itself. In a hierarchical structure, each node has the power to control access, but there are no incentives for proactive collaboration. This means that the time it takes to complete a process increases linearly with the number of levels in the hierarchy. This finding suggests that merely reducing the approval time at individual nodes is unlikely to fundamentally resolve the issue; instead, a transition to a parallel, collaborative process structure is necessary.

#### 2.4.2 Difficulties in departmental collaboration, with silo effects present

Hospitals feature complex internal structures. Influenced by long-standing siloed management, functional departments maintain distinct boundaries. Staff often remain confined to their specific duties, lacking initiative for cross-departmental collaboration. Crucially, this fragmented work pattern persists due to the absence of institutionalized coordination mechanisms (13, 14) which leads to inefficiencies and delays in project completion across departments. Research indicates (15) that 76.3% of administrative staff perceive lengthy processes between departments, while 68.9% consider cross-departmental collaboration inefficient. Therefore, establishing interprofessional collaboration (IPC) mechanisms is an effective approach to breaking down silos within healthcare organizations and enhancing administrative responsiveness (16). However, most public hospitals have yet to establish routine administrative collaboration systems comparable to clinical MDTs. Interdepartmental collaboration lacks standardized processes, leading to buck-passing and redundant work in overlapping functional areas. This forces clinical staff to repeatedly coordinate across departments, significantly increasing communication costs. During the patient discharge settlement process, which involves multiple departments including clinical services, billing, medical insurance, and medical records, information gaps and disjointed workflows between departments result in an average discharge settlement time exceeding 1 h. This is significantly longer than the 20 min average observed in Singapore's public hospitals (7).

The theory of collaborative governance suggests that the effectiveness of cross-departmental collaboration depends on institutionalized coordination mechanisms rather than the willingness of individuals to cooperate. The fundamental cause of coordination difficulties among the hospital's departments is not a subjective unwillingness on the part of staff to cooperate, but rather a lack of institutionalized evaluation mechanisms, deliberation rules, and procedural standards for collaborative behavior. This finding resonates with the research by Wei et al. (14) on collaborative linkage mechanisms, further clarifying the central role of administrative MDTs as institutionalized vehicles for collaboration in breaking down the “silo effect,” and providing a theoretical basis for subsequent reform practices.

#### 2.4.3 Barriers to information exchange and inefficient data utilization

Despite ongoing advancements in public hospital IT infrastructure, current paperless operations primarily involve simple digital migration of traditional workflows, failing to leverage big data and AI for process reengineering and intelligent transformation (17). Ineffective connectivity between systems—such as HR, finance, logistics, and medical operations—coupled with inconsistent data standards and incompatible system interfaces, forces frontline administrators to repeatedly input data across multiple platforms. This not only increases operational burdens but also heightens the risk of data errors. Research by Qian et al. (18) indicates that while information mechanisms should serve as the foundation for collaborative governance, practical implementation often suffers from fragmented standards systems, overly complex process designs, and difficulties in interface compatibility. These issues reflect a significant lag in the supply of technical specifications. The failure to effectively integrate platform functions with departmental workflows results in delayed information updates, inconsistent data formats, and diminished practical value, thereby creating new “data silos.”

At the same time, separating operational workflows from data flows makes it harder to share and integrate hospital operational data. This prevents managers from gaining timely, comprehensive insights into the hospital's actual operational landscape. Consequently, major management decisions rely on past experience or fragmented data, making it difficult to conduct scientific analysis and implement precise measures based on comprehensive data. This severely constrains the advancement of refined management practices in public hospitals (19). From an international comparative perspective, the high level of data integration achieved by Japan's public hospitals is closely linked to its nationally unified medical information standards system, long-term and stable investment in information technology, and institutional design that integrates administrative and clinical functions. However, when adapting such experiences to the local context, it is necessary to carefully assess the practical differences in China's public hospitals regarding information system development pathways, data ownership, and departmental interests. Simply emphasizing the technical aspect of “interoperability through a single database” while neglecting the establishment of institutional coordination mechanisms risks falling into the trap of “technological determinism” and makes it difficult to truly break down the data barriers that exist in practice (20). Therefore, when promoting data integration, China's public hospitals must simultaneously advance standardization, clarify responsibilities and authorities, and establish incentive mechanisms, thereby organically combining technological empowerment with institutional restructuring.

Theories of organizational process reengineering emphasize that the application of information technology should serve the fundamental restructuring of processes, rather than simply replacing manual operations. This study found that the current development of information systems in public hospitals generally remains at the stage of “digitization” rather than “reengineering,” which is precisely the underlying cause of the persistent problem of “data silos.” Even with advanced information systems, without cross-departmental data governance mechanisms and standardized business process guidelines, the technology itself cannot automatically achieve process optimization, which is essential for overcoming the inefficiencies caused by data silos in public hospitals.

#### 2.4.4 Misaligned service mindset and management orientation

The core responsibility of administrative and logistical departments is to serve clinical operations and support frontline staff. However, in practice, some management systems exhibit a pronounced tendency to “prioritize control over service.” At its root, this issue stems from a shortage of multidisciplinary talent within administrative and logistical teams—individuals who understand medicine, excel in management, and possess technical expertise—resulting in a mismatch between professional capabilities and job requirements (21, 22). Administrative personnel typically fall into three categories: first, frontline clinical medical staff who transition into administrative roles after reassignment; second, individuals who pursue administrative work aligned with their professional background; and third, administrators whose expertise does not match their field but possess relevant experience. Except for those transitioning from clinical frontline positions, the remaining administrative staff exhibit certain deficiencies in professional competency. A study on administrative personnel competency revealed (23) that 52.4% of administrative incidents exhibited a lack of first-contact responsibility. Administrative staff demonstrated an execution capability of 83.3%, project management capability of 76.2%, and adaptability of 75.4%, reflecting core bottlenecks in administrative effectiveness. Additionally, 46.8% of administrative staff exhibited a deficient sense of responsibility, and 44.4% demonstrated a lack of a comprehensive perspective, underscoring the deficiencies associated with unclear job responsibilities, insufficient professional alignment, and misdirected service awareness in administrative positions. Devoid of clinical perspective, certain functional departments formulate regulations primarily from the standpoint of administrative convenience and liability avoidance, neglecting the practical feasibility and efficiency of these systems in clinical settings, which can lead to increased frustration among clinical staff and hinder patient care. This results in cumbersome reporting requirements, redundant record-keeping, and frequent evaluations, which inadvertently increase non-clinical burdens on frontline medical staff. This misalignment of management roles forces medical staff to devote substantial energy to administrative tasks, crowding out time for patient care and research, thereby deviating from the hospital's original mission of “patient-centered” service. Research indicates that (24) clinical staff at this hospital spend an average of over 2 h daily on administrative tasks, significantly exceeding the 1-h average for medical personnel in UK NHS public hospitals. Hospital administrative processes suffer from issues such as “cumbersome procedures, poor interdepartmental coordination, and delayed information transmission.” This excessive non-clinical workload has become a major factor impacting clinical work efficiency.

When management systems shift from being tools to ensure clinical services to becoming barriers for departments to avoid risks, administrative management deviates from its original purpose of serving core business operations. Compared to the study by Minmin et al. (22) on the administrative MDT model, this study not only identifies manifestations of a misaligned service mindset but also traces its root causes to the professional composition and role positioning of administrative staff. The study found that administrative staff lacking a clinical perspective tend to prioritize managerial convenience when formulating policies. This finding underscores the importance of cultivating versatile administrative talent and establishing mechanisms for administrative-clinical integration, thereby providing a problem-oriented framework for the implementation of multi-skilled training and clinical rotation programs in future reforms.

### 2.5 Practical exploration of administrative processes in public hospitals

Facing the mismatch between administrative efficiency and high-quality development demands, a large public hospital in an eastern coastal city of China, grounded in its operational realities and adopting a problem-oriented approach, has undertaken systematic administrative process reforms. By breaking down hierarchical barriers, reshaping service workflows, and integrating functional structures, we implemented systematic administrative process reforms. Specific, actionable plans were developed for core areas including the establishment of administrative MDTs, the reengineering of multi-functional roles, and the restructuring of the logistics department. The hospital has established a new administrative management model characterized by smooth operations and timely responses, achieving significant practical results. Meanwhile, the hospital drew extensively on advanced experiences from countries such as Singapore and Japan during its reform process (6–8), integrating these insights with the operational realities of Chinese public hospitals to foster localized innovations. This approach has shaped an administrative reform pathway with distinctive Chinese characteristics. In drawing on international experience, the hospital did not simply adopt practices from other countries; instead, through critical comparison, it identified the key areas for localization. For example, the one-stop administrative service model of Singapore's public hospitals relies on a nationwide unified data platform and highly standardized service processes. When adopting this model, the hospital took into account the reality of China's fragmented hospital information systems and weak cross-departmental coordination mechanisms which necessitated the development of tailored solutions to integrate these systems effectively and improve coordination among departments. It prioritized the parallel advancement of offline “one-window processing” and online “mobile processing,” using spatial integration to compensate for the lack of data sharing. Meanwhile, the clinical rotation system for administrative staff in Japanese public hospitals is built upon a relatively mature personnel exchange mechanism and a long-term training system. When adopting this model, the hospital adopted a “pilot-first, phased implementation” approach, prioritizing the exploration of multi-skilled training in roles such as logistics management and patient services to avoid implementation difficulties caused by institutional disparities. This localized adaptation, based on comparative analysis, maintains the fundamental principles of international best practices while guaranteeing the practicality and longevity of reform initiatives within the institutional framework of China's public hospitals. This study's practical exploration was conducted through descriptive analysis of a single case and did not involve long-term tracking of the implementation outcomes of the administrative MDT collaboration mechanism. Future research should conduct long-term tracking and quantitative validation of the implementation outcomes of this mechanism. Concurrently, comparative analysis with administrative reform models from other public hospitals in China is necessary. Through continuous optimization, the universality of the administrative reform model can be enhanced, making it more adaptable and effective across various public hospitals in China.

#### 2.5.1 Breaking barriers: establishing a routine administrative MDT collaborative governance mechanism

To address the pain points of poor coordination and frequent buck-passing among functional departments, the hospital drew inspiration from the MDT approach in clinical settings. It also referenced Japan's cross-departmental collaboration assessment model in public hospitals and Singapore's one-stop collaborative service experience. The hospital set up a standardized, multi-tiered system for managing administrative collaboration. It made specific operational plans in four areas: institutional design, organizational structure, operational processes, and assessment supervision. This mechanism breaks down departmental administrative boundaries and achieves closed-loop management of clinical requests. This team-based management model has been proven to effectively reduce organizational complexity and enhance the efficiency of resolving complex issues (25).

The theory of collaborative governance emphasizes that institutionalization is key to transforming cross-departmental collaboration from *ad hoc* cooperation into routine operations. The hospital's administrative MDT mechanism is a practical embodiment of this theoretical logic: it institutionalizes collaborative work through the establishment of an administrative MDT management office; clarifies resolution pathways and timelines for different types of issues via tiered handling standards; and integrates collaborative behavior into the performance evaluation system through assessment and oversight mechanisms. This institutional design shifts collaboration from relying on “personal connections” to relying on “mechanisms,” effectively addressing the challenges in interdepartmental collaboration identified in the preceding analysis such as inconsistent communication and unclear responsibilities among departments.

Based on the difficulty and scope of issues, the institution has established a three-tiered problem-solving channel to enable tiered and categorized handling. It has clearly defined the operational procedures, responsible entities, processing timelines, and assessment criteria for each tier:

##### 2.5.1.1 Core operational protocol for administrative MDT

Organizational structure design: Establish an Administrative MDT Management Office as the daily operational management body, located within the hospital president's office and staffed with dedicated personnel. This office will be responsible for coordinating all aspects of the Administrative MDT, including issue collection, task classification and assignment, progress tracking, and performance evaluation, to ensure the routine operation of the Administrative MDT.

Problem collection mechanism: Establish a dual-channel problem collection system combining online and offline approaches. Online, leverage the hospital's OA system and mobile app to launch the “New Li You Process Optimization Suggestion Collection” initiative, enabling clinical departments to submit issues at any time. Offline, proactively gather clinical needs through administrative rounds, clinical symposiums, and other methods to achieve comprehensive and real-time problem collection.

Tiered processing standards: Define the criteria for classifying issues into three tiers: Tier 1 issues: Simple problems resolvable by a single department, with a processing deadline of 1 business day. Tier 2 issues: Moderately complex problems involving two to three departments, with a processing deadline of three business days. Tier 3 issues: Persistent problems spanning three or more departments, with a processing deadline of seven business days. Extensions may be requested for exceptionally complex cases, but the maximum processing time shall not exceed 15 business days.

Performance evaluation and oversight mechanism: Incorporate the implementation of administrative MDT work into the annual performance evaluations of all departments. Evaluation metrics shall include issue response rate, timely completion rate, and clinical satisfaction. Evaluation results shall be directly linked to departmental performance bonuses and the recognition of department heads for excellence, thereby compelling all departments to proactively collaborate and coordinate their efforts.

##### 2.5.1.2 Specific operational process of the three-tier resolution channel

Decision-making level: An Administrative MDT Committee was formed, led by hospital leadership and involving heads of administrative and logistical departments. Leveraging regular functional department meetings, this committee conducts specialized research on long-standing, cross-departmental “intractable” issues, achieving on-the-spot resolutions with strict enforcement. This clarifies decision directions and assigns responsibility.

Project level: Tailored to China's national context, specialized MDT task forces are formed for high-complexity initiatives with clear objectives—such as improving national assessment metrics or Diagnosis Related Groups (DRG) payment reforms. These teams transcend departmental boundaries, assembling core professionals from various units for centralized operations. They implement end-to-end management, conduct quarterly progress reviews and corrective actions to ensure efficient project advancement.

At the operational level: Implement a joint administrative ward round system. Form inspection teams led by hospital leadership and involving relevant functional departments to engage directly with clinical departments on the front lines and address clinical needs in real time. Issues that cannot be resolved on-site—such as slow equipment repairs or cumbersome system operations—are added to a supervision list. This list specifies responsible parties and deadlines, employs a ticketing system for closure, and ensures every clinical request receives a resolution and a response (14).

This model achieves full-process closed-loop management through a data platform and task oversight lists, yielding remarkable results. According to descriptive data (26), from January 2021 to December 2025, the hospital saw steady growth in core operational metrics including outpatient visits, discharges, and surgical procedures. It ranked first among integrated Chinese and Western medicine hospitals in the province (a hospital type unique to China that integrates modern medical and traditional Chinese medical diagnostic and treatment methods) in national performance evaluations, with its Case Mix Index (CMI) ranking improving by eight positions. These changes in the aforementioned indicators coincided with the implementation of the administrative MDT collaborative governance model. Institutionalized coordination mechanisms can effectively reduce organizational complexity and improve the efficiency of resolving cross-departmental issues. Compared with similar studies both domestically and internationally, this study presents a comprehensive operational framework for administrative MDTs—covering institutional design, operational processes, and performance evaluation and oversight—rather than merely advocating for the concept, thereby providing a practical implementation model for other public hospitals.

#### 2.5.2 Process reform: driving administrative service process reengineering with “convenience” at its core

Aligning with Zhejiang Province's “One-Visit-Maximum” government service standard, the hospital shifted administrative service focus downward, addressing bottlenecks and pain points for medical staff and patients. Through spatial integration and digitalization, administrative processes were optimized and reengineered, significantly enhancing efficiency and service experience. First, implementing “one-stop service windows” optimized patient processes: Supporting System Optimization: Launched an intelligent queue management system enabling online appointment scheduling and offline queue notification for patient services, eliminating window queues. Streamlined the electronic diagnostic certificate process, synchronizing certificates issued by physicians in real-time to the Patient Service Center, eliminating the need for patients to visit departments for printing. Integrated interfaces across business systems, enabling frontline staff to “operate multiple systems through a single screen,” significantly boosting processing efficiency. Dispersed outpatient service centers were restructured to eliminate functional silos between consultation guidance, medical record stamping, refund verification, and medical insurance policy inquiries. Service resources were consolidated, and staff received cross-functional training to achieve “single-window acceptance and comprehensive processing.” Develop training manuals and conduct regular training sessions for frontline staff, covering job-specific knowledge, service etiquette, and emergency response procedures. Establish a performance evaluation system for multi-skilled staff, with service efficiency and patient satisfaction as core metrics, to incentivize continuous improvement in service capabilities. Combined with an intelligent queue management system, streamlined electronic diagnostic certificate processes, and integrated business system interfaces, According to publicly available data, the hospital reduced average patient wait times from 10–20 min to just 2–5 min, and the patient experience has improved. However, since this data does not account for potential confounding factors such as changes in patient volume or adjustments to the number of staff at service counters during the same period, the results presented are purely descriptive. Secondly, we are advancing “mobile processing” to streamline internal administrative workflows. Incorporating “administrative process reform” into the hospital's development initiative, the upgraded OA (Office Automation) system migrated high-frequency administrative tasks—such as leave requests, operational approvals, and maintenance reports—to a mobile app. We conducted research to identify 28 high-frequency administrative tasks that clinical medical staff handle, including leave requests, supply requisitions, operational reports, maintenance requests, and expense reimbursements. We standardized the processing procedures, required materials, and approval stages for all tasks, compiling the Mobile Processing Guide. Leveraging the upgraded OA system and hospital mobile app, we achieved full online implementation for all procedures. This enables administrative approvals to be handled “on-the-go, anytime, anywhere.” Simultaneously, the hospital established and strictly enforced a first-contact responsibility system and a time-limited completion system, which ensures that patient inquiries are addressed promptly and that all procedures are completed within a specified timeframe to enhance overall efficiency. The first staff member to receive an inquiry or application is designated as the primary responsible party, who tracks the entire processing progress. Clear deadlines are set for each approval stage, compelling administrative staff to improve efficiency. Real-time approval progress is displayed on the mobile app, allowing clinical staff to check at any time, ensuring transparency and traceability throughout the approval process. This effectively resolves the frontline clinical issues of “difficulty finding the right person” and “slow processing,” earning unanimous praise from medical staff (1). For non-core matters, a “parallel collaborative approval” system is implemented. For example, departmental material requisitions can be submitted simultaneously to both the Logistics Management Center and the Finance Department for approval, reducing the processing time from three working days to one. For urgent matters, a “green approval channel” is established. For instance, requests for emergency medical equipment repairs can be submitted directly to hospital leadership, enabling “immediate approval and immediate handling.” Third, the hospital is implementing a large-department reform for logistics to build an integrated support system. The hospital restructured its logistics organization through large-department reform to address longstanding issues, such as fragmented management, dispersed responsibilities, and passive responses. By consolidating functions and unifying authority, it established an integrated, refined logistics support system. Fourth, the hospital consolidated functional structures to achieve centralized management. Dispersed departments such as security, general affairs, and infrastructure were dissolved and reorganized into a unified Logistics Management Center with consolidated authority and responsibilities. This center centrally coordinates logistics operations, including security and fire safety, environmental sanitation, energy consumption monitoring, building automation, and material distribution. A unified smart logistics dispatch platform was established, driving the transformation of logistics support from extensive, experience-based management to refined, professional governance (21). To avoid overlapping functions and authority gaps, make sure that each department in the Logistics Management Center knows what its main duties are, what its operational limits are, how it will work with other departments, and how it will measure its performance. For instance, the Supply Distribution Department is in charge of buying, storing, and distributing all medical and administrative supplies throughout the hospital. The Equipment Maintenance Department is in charge of fixing and maintaining all medical and office equipment. Establish a smart logistics dispatch platform integrating all operational data—including security monitoring, energy consumption tracking, equipment performance, maintenance requests, and supply distribution—to enable visualized, intelligent management of logistics operations. The platform interfaces with the hospital mobile app, allowing clinical departments to submit maintenance requests anytime. The system automatically assigns tasks to designated technicians, who receive real-time notifications and report progress updates. Resulting in closed-loop management of the maintenance workflow. Fourth, transform service models to achieve proactive support. Shift logistics services from traditional “reactive maintenance” to “proactive service.” Establish a regular clinical inspection system where logistics staff visit clinical departments to proactively inspect equipment and facilities, promptly identifying and eliminating potential hazards for early detection and resolution. Through this large-department reform, the hospital established a unified, efficient, and professional logistics operations command center, significantly enhancing service response speed and support capabilities.

The theory of organizational process reengineering emphasizes that process optimization should aim for fundamental improvements rather than piecemeal fixes. Through its “one-stop service” initiative, the agency has broken down the functional silos between service windows, achieving a fundamental shift from “queuing at multiple windows” to “single-window processing”; “Mobile Processing” has migrated traditional offline approvals to an online platform, supplemented by a first-case-responsibility system and a time-limited completion system, achieving a shift from a “passive waiting” to an “active follow-up” model; the “Centralized Logistics System” has consolidated dispersed functions and established a unified dispatch platform, achieving a structural shift from “multi-headed management” to “unified command.” These three reforms center on the needs of service recipients and reconstruct workflows rather than simply optimizing existing ones, collectively embodying the core philosophy of process reengineering. Compared to Xiaoying's (24) research on the standardization of administrative processes, the uniqueness of this study lies in revealing the interactive relationship between process reengineering and digital infrastructure development. While advancing “mobile processing,” the court simultaneously established a first-responsible-officer system and a time-limited resolution system, embedding institutional constraints into the technical platform; while advancing the “logistics department system” reform, it simultaneously built a smart logistics dispatch platform, combining functional integration with technological empowerment. This experience demonstrates that the success of process reengineering depends not only on technological upgrades but also on the deep integration of institutional design and technological application.

### 2.6 Challenges and potential risks in implementation

The implementation of administrative process reforms may encounter various challenges and potential risks, which warrant close attention.

#### 2.6.1 Potential implementation costs

Measures such as establishing an administrative MDT management office staffed with dedicated personnel, developing or upgrading OA systems and mobile apps, conducting training on multi-skilled roles, integrating logistical functions, and building a smart scheduling platform all require corresponding financial investment and human resource allocation. However, publicly available information has not yet disclosed specific amounts or changes in staffing levels; hospitals of different sizes and with varying levels of IT infrastructure should conduct cost estimates based on their own conditions when replicating this approach. Furthermore, the routine operation of the administrative MDT requires ongoing evaluation, supervision, and coordination, which also generates implicit management costs that can vary significantly depending on the size of the hospital and the complexity of the MDT's functions.

#### 2.6.2 Potential implementation challenges

The establishment of cross-departmental collaboration mechanisms may encroach on the boundaries of middle managers' authority and responsibilities, and some functional departments may resist such changes out of concern that their power will be reduced, performance evaluation pressures will increase, or their workflows will be subject to external interference. The transition of administrative staff from a “control” to a “service” role requires a lengthy adjustment period, and there may be conflicts between the work habits established under the existing hierarchical system and the new culture of collaboration. Furthermore, trust in the administrative MDT mechanism among clinical departments needs to be built gradually; in the early stages, there may be a lack of cooperation or reluctance to report issues.

#### 2.6.3 Potential unintended consequences

First, administrative MDT processes tend to become bureaucratic. A collaborative mechanism, initially intended to dismantle departmental barriers, may gradually evolve into additional layers of approval and paperwork procedures. For example, overly cumbersome designs in stages such as tiered task assignment, progress tracking, and closed-loop feedback may actually increase the burden on administrative staff and clinical departments, leading to bureaucratic formalism. Therefore, implementing units should focus on streamlining processes and regularly assess the time costs associated with each stage. Second, there is a tendency toward superficial compliance with performance metrics. When indicators such as clinical satisfaction, on-time completion rates, and issue response rates are directly linked to departmental evaluations and bonuses, organizations may selectively report easily resolved issues, lower clinical expectations, or use technical means to manipulate data—all while actual service quality remains unchanged. There is a risk that, in pursuit of on-time completion rates, complex issues may be downgraded or processed prematurely only to be continued privately afterward. Therefore, it is recommended that implementing units establish multi-dimensional monitoring mechanisms—such as anonymous clinical satisfaction surveys, random follow-ups, and monitoring of outliers in process durations—to identify genuine changes in service quality underlying the metrics.

### 2.7 Reflections on reform pathways for administrative processes in public hospitals of eastern coastal cities, China

Administrative process reform in public hospitals in China's eastern coastal cities is not merely a matter of small adjustments to local procedures. It involves a fundamental transformation of management models and a restructuring of systems that touches upon the core of governance. Such reform must be grounded in the public welfare and developmental realities of public hospitals, adhere to scientific reform principles, and construct a systematic and targeted reform pathway to ensure tangible outcomes. However, reform pathways derived from a single case study are not universally applicable. It is necessary to clarify under what organizational, regional, or policy contexts they are most likely to succeed, as well as under what circumstances significant adjustments are required, to provide truly meaningful insights for other public hospitals.

#### 2.7.1 Conditions for the transferability of reform pathways

Regarding the conditions for applicability, the first consideration is the size and complexity of the organization. This method works well for public hospitals with more than 1,000 beds and more than a million outpatient visits each year. Such hospitals typically have relatively comprehensive departmental structures and complex administrative processes, making the marginal benefits of reform particularly significant. For secondary and lower-level hospitals, the administrative MDT collaboration mechanism and process reengineering concepts may be selectively adopted, but the organizational hierarchy must be simplified accordingly. Second, attention must be paid to the regional economic and IT infrastructure. The case hospital is located in a coastal city in eastern China, where the economic foundation is strong and investment in IT is well-supported. Public hospitals in economically developed regions can directly reference the digital development plan outlined in this approach, while hospitals in less developed regions or those with weak IT infrastructure are advised to prioritize the implementation of offline “one-stop service” and basic process reengineering, and gradually introduce tools such as data middleware and mobile BI once conditions are ripe. Third, the policy environment is also critical. This pathway aligns closely with national policy directions such as the promotion of high-quality development in public hospitals and medical insurance payment reforms [DRG/Data Integration Platform (DIP)]. In regions where healthcare reform policies are rigorously enforced, resistance to reform is minimal; conversely, if there is a lack of support from higher-level policies or local implementing regulations, it is necessary to first secure policy authorization or adjust the priority of the reform. Fourth, personnel and organizational culture also influence the implementation of this reform path. This approach requires administrative staff to possess a certain willingness to learn and a sense of collaboration. If departmental interests within the hospital are deeply entrenched and staff resistance to change is significant, efforts to shape the organizational culture and provide training should be undertaken first, or pilot programs should be launched in departments with a stronger foundation for collaboration.

Simultaneously, one should focus on circumstances that necessitate substantial modifications. First, for small hospitals or single-specialty hospitals with fewer than 500 beds, the three-tier resolution process for administrative MDTs may be overly complex. It is recommended to simplify this into a two-tier mechanism for handling single-department and cross-departmental issues, and to merge the administrative MDT committee with the hospital administration meetings. Second, for hospitals with severely lagging IT infrastructure—specifically those that have not yet established an OA system or a basic Hospital Information System (HIS) system—they should not directly emulate “mobile processing” or data middleware construction. Instead, they should prioritize completing basic IT coverage and the digitization of core business processes. Third, for public hospitals with multiple campuses or those under group management, the large-department reform outlined in this pathway needs to be further expanded into a two-tiered structure linking campuses with headquarters, incorporating coordination mechanisms and information synchronization modules between campuses. This pathway primarily focuses on internal reforms within single-site hospitals; if applied to tightly integrated medical consortiums or medical communities, additional design considerations are required regarding cross-institutional data sharing, unified service standards, and collaborative performance evaluations, and it cannot be directly applied without addressing the complexities of inter-hospital collaboration and the need for standardized protocols across different institutions.

Therefore, when other public hospitals adopt this approach, it is recommended that they first assess their suitability across five dimensions—scale, financial foundation, level of IT infrastructure, policy support, and organizational culture—and select the reform modules that best align with their conditions for pilot implementation before gradually expanding the initiative.

#### 2.7.2 Fundamental principles of reform

The reform process centers on clinical needs and prioritizes grassroots perspectives, consistently adhering to the core principle that “administration serves clinical practice.” It eliminates excessive administrative interference and constraints on clinical diagnosis and treatment, making the reduction of non-clinical workloads for medical staff a key reform objective. Modern hospital management theory emphasizes that effective support for medical staff and mitigation of professional burnout are prerequisites for improving healthcare service quality (27). Reforms must resolutely streamline redundant approval procedures and eliminate ineffective performance metrics, enabling medical personnel to refocus on core healthcare services.

Systematic Planning and Coordinated Advancement. Treating institutional restructuring, authority realignment, and process reorganization as an integrated project, we will implement a holistic design with phased execution. This approach will rationalize vertical and horizontal management relationships, prioritize cross-departmental coordination and vertical integration, and avoid governance pitfalls where “old walls are torn down only to erect new barriers” (19), ensuring the reform's comprehensiveness, synergy, and sustainability.

Technology-Driven, Data-Empowered. Digital transformation must transcend merely replicating traditional processes online. Centered on a data middle platform, it should unify data standards and business logic across systems, driving deep integration of information technology with administrative management. This enables automated and intelligent transformation of business processes, leveraging comprehensive data to support scientific decision-making. Digital technology must play a central role in enhancing administrative efficiency and achieving refined management by streamlining workflows, automating routine tasks, and providing real-time data analytics to inform decision-making (26).

Prioritize Efficiency While Ensuring Compliance. While streamlining administration and optimizing processes, strengthen internal control mechanisms. Embed internal control requirements into every stage of administrative processes through intelligent means to achieve precise risk prevention and control. This ensures that while administrative processes enhance responsiveness, management quality and operational standards are maintained, achieving dual improvements in efficiency and compliance (28, 29).

This approach involves drawing on international best practices and adapting them to local contexts. The core of the Singaporean model lies in service integration achieved through “standardized processes and a unified platform.” This approach is suitable for scenarios with a solid IT infrastructure and a strong need for cross-organizational collaboration, but it places high demands on data standards and interdepartmental coordination. The core of the Japanese experience lies in the collaborative awareness fostered by “institutional embedding and personnel integration,” which is appropriate for situations where administrative management and clinical work are deeply intertwined, although it requires long-term support from talent development and evaluation mechanisms. Additionally, the core of the British experience focuses on cost control achieved through.

The case hospital featured in this study actively drew upon the advanced experiences of administrative reforms in public hospitals in countries such as Singapore, Japan, and the United Kingdom during its own reform process. It conducted an in-depth analysis of the core logic and operational methods of these reforms, but did not blindly copy them. Instead, it adapted and innovated these approaches to suit the context of China's public hospitals by taking into account their public welfare nature, management systems, and operational realities. The hospital chose to use administrative MDTs to resolve coordination challenges, reengineered service efficiency through multi-functional roles, and consolidated resource support through a centralized logistics department. This approach has forged a path for administrative reform in public hospitals with Chinese characteristics—one that combines an international perspective with local feasibility.

#### 2.7.3 Core strategies for reform

Based on practical exploration and theoretical analysis, reforming public hospital administrative processes requires establishing a systematic framework with three pillars: system restructuring, technological empowerment, and talent development. This framework drives the modernization of administrative management systems.

Four key components achieve system restructuring. First, dismantle boundaries and shorten chains to build a collaborative management system. During implementation, focus on eliminating departmental silos and shortening management chains to systematically restructure the administrative framework. This consolidates functional department synergies and enhances collaborative management effectiveness. Second, optimize organizational structures by advancing large-department reforms. This involves streamlining upper-level management structures, consolidating departments with similar functions and related operations, and establishing dedicated cross-functional units such as operational management offices and patient service offices. To avoid overlapping functions and authority vacuums, each institution has clearly defined core responsibilities. Third, a standardized administrative MDT model is put into place. Institutionalize and normalize the administrative MDT collaborative governance mechanism by establishing fixed deliberation rules, decision-making processes, and execution oversight mechanisms. This enables efficient coordination and resolution of cross-departmental issues, breaking down siloed operations. Fourth, reconstruct the authority and responsibility operation system. Clarify the boundaries of responsibilities for each department and position, establish a system of authority and responsibility lists, and optimize the traditional sequential approval model into a parallel collaborative processing model. Reduce approval levels while strictly implementing the first-contact responsibility system and time-limited completion system, clearly defining processing responsibilities and deadlines to enhance administrative efficiency (13). Fifth, integrate international collaboration experiences (30). Drawing on Japan's experience of deep integration between administration and clinical operations in public hospitals, establish cross-departmental communication mechanisms between administrative and clinical staff. Regularly convene administrative-clinical symposiums to ensure administrators promptly understand clinical needs. Learning from Singapore's experience of cross-departmental collaborative assessment in public hospitals, incorporate the implementation of collaborative work into the performance evaluations of all departments to strengthen interdepartmental collaboration awareness.

To empower technology, it is crucial to dismantle barriers, pursue intelligent upgrades, and establish a digital management system. By leveraging digital transformation as a catalyst, we must completely dismantle information silos to achieve data integration, process optimization, and intelligent decision-making, making digital technology the core driver for enhancing administrative efficiency. First, we will build an integrated data middle platform and a Mobile Business Intelligence (BI) system. Unify data standards and interface specifications across all hospital business systems to establish an integrated data hub. This enables single-source data collection and multi-party sharing, fundamentally eliminating redundant reporting and achieving “one source, multiple uses.” Leveraging the Mobile BI system facilitates real-time data analysis and visualization, providing robust data support for management decisions. Secondly, promote the digitization and automation of administrative processes. Transition routine administrative tasks—such as paper approvals, material requisitions, and report statistics—to electronic formats. Implement workflow engines to automate process circulation and enable intelligent approvals, reducing manual intervention and boosting processing efficiency. Thirdly, explore artificial intelligence and large-model applications. Develop an intelligent assistant for hospital administration to support contract review, policy consultation, process guidance, and risk alerts, elevating the intelligence of administrative management. Create a visual management dashboard to display real-time, multi-dimensional operational metrics, providing real-time data analysis, risk alerts, and decision support for managers at all levels (26). Fourth, promote regional medical data sharing and coordination. Drawing on the experience of Singapore's national unified medical data platform (6), under the coordination of regional health authorities, advanced data sharing and coordination among public hospitals, primary healthcare institutions, medical insurance departments, disease control centers, and other relevant units. This will allow different institutions to handle administrative tasks—like medical insurance payments and registering for medical treatment outside their area—making administrative services more convenient.

We achieve talent development, by enhancing capabilities, shifting mindsets, and building a specialized service-oriented team. The implementation of systems and technologies ultimately relies on people. Through tiered training, incentive mechanisms, and cultural shaping, a professional, multi-skilled administrative management team should be cultivated to drive the shift in administrative philosophy from “management” to “service.”

First, establish a tiered and categorized talent development mechanism: Conduct specialized training in refined management, data analysis, and strategic leadership for frontline, mid-level, and senior administrative managers to enhance their professional capabilities. Actively recruit multidisciplinary talent with backgrounds in medicine, management, and information technology to optimize the administrative team structure (23). Second, strengthen clinical practice exposure: Establish mechanisms for administrative staff to rotate through clinical departments and participate in cross-departmental projects. This immerses administrators in frontline clinical settings, deepens their understanding of practical clinical needs, cultivates their ability to solve complex problems, and instills a core mindset of “serving clinical departments.” Third, refine performance evaluation and incentive mechanisms: Center assessments on tangible outcomes and service recipient satisfaction. Introduce internal satisfaction surveys of administrative departments by clinical departments and frontline staff, incorporating survey results as key metrics for evaluating administrative quality and efficiency. Establish a performance-oriented approach where “service quality determines performance, and effectiveness dictates rewards and penalties” (31). Fourth, shape a service-oriented administrative culture: Make proactive clinical service and problem-solving second nature for administrative staff by making administrative rounds and clinical visits a regular part of their jobs. Strengthen cultural development within administrative departments, reinforcing service awareness, collaborative spirit, and efficiency consciousness to foster a positive work atmosphere centered on “serving clinical departments and supporting frontline operations” (32). Fifth, establish an international exchange and learning mechanism. Forge cooperative relationships with renowned public hospitals in countries such as Singapore and Japan. Regularly dispatch administrative personnel for overseas exchanges to absorb advanced management concepts and operational methodologies. Simultaneously, invite international healthcare management experts to conduct training and provide guidance at the hospital, thereby enhancing the international perspective and professional capabilities of administrative staff (30).

## 8 Conclusion

The high-quality development of China's public hospitals is a core requirement for building the national healthcare service system, and optimizing administrative processes is crucial for enhancing hospital management efficiency and ensuring healthcare service quality. Constrained by traditional management models, current public hospital administrative processes suffer from prominent issues such as lengthy approvals, coordination difficulties, information bottlenecks, and service misalignment, necessitating urgent transformation. Therefore, the reform of administrative processes in Chinese public hospitals must consistently adhere to the principles of clinical-centered planning, systematic design, technology-driven implementation, and balanced efficiency with compliance. Focusing on system restructuring, technology empowerment, and talent development, it should break down departmental silos, shorten management chains, eliminate information barriers, and enhance personnel capabilities. This will drive the transformation of the administrative management system from a traditional hierarchical structure toward a collaborative, digital, and service-oriented model.

At the theoretical level, this study expands upon and deepens the theories of bureaucracy, collaborative governance, and organizational process reengineering. The research reveals that, in a traditional environment characterized by high stability and a clear division of labor, the bureaucratic system offers efficiency advantages in the administrative context of public hospitals. However, against the backdrop of increasingly diverse clinical needs and more frequent cross-departmental collaboration, the bureaucratic principles of division of labor and hierarchical control are prone to evolve into departmental barriers and process rigidity, which can hinder effective patient care and decision-making in public hospitals. This discovery broadens the explanatory framework of bureaucratic theory in professional organizations. Furthermore, the study introduces collaborative governance theory from the macro-policy level into the hospital administrative context, highlighting how this theory can facilitate improved communication and cooperation among departments to overcome the identified barriers. By designing administrative MDTs as institutions, it finds the main conditions that need to be met in order to turn “*ad hoc* cooperation” into “routine operation.” This gives collaborative governance theory a way to work at the meso-level. Furthermore, building on organizational process reengineering theory, the study proposes a process reengineering logic of “parallel technical empowerment and institutional restructuring.” It reveals that technical upgrades alone cannot achieve fundamental improvements; rather, institutional constraints must be embedded within technical platforms, and functional integration must be combined with technical empowerment to ensure that both the technology and the organizational structure work synergistically to enhance overall performance. This insight enriches the theoretical framework of process reengineering within the context of healthcare organizations.

Practices in reform at a public hospital in a coastal city in eastern China demonstrate that, while implementing measures such as an administrative MDT collaborative governance mechanism, process reengineering for multi-functional positions, and a major restructuring of the logistics department, the hospital observed a series of positive changes in breaking down traditional management barriers, enhancing administrative efficiency, and reducing non-clinical administrative burdens. The hospital's overall operational performance indicators also showed improvement. Meanwhile, the core principles and reform pathways of this hospital offer valuable insights for economically underdeveloped regions in China. However, their implementation requires localization tailored to local healthcare system characteristics, resource conditions, and policy environments, avoiding simplistic replication. Moreover, its core principles of collaborative governance, process reengineering, and digital empowerment—through adaptive adjustments and collaborative research—can facilitate policy dialogue and knowledge sharing for public hospital reforms in other low- and middle-income countries. These approaches also hold significant reference value for enriching empirical research on organizational transformation and administrative efficiency in public hospitals within the international health policy and management field.

This study offers valuable insights for policy-making and hospital management practices. First, the findings reveal that the underlying obstacles to administrative process reform in public hospitals lie not only in operational inefficiencies but also in the lack of institutional coordination mechanisms and misalignments in the allocation of authority and responsibility. This insight suggests that, when promoting management transformation in public hospitals, policymakers must go beyond operational requirements such as “streamlining approvals” and “optimizing processes” and instead prioritize the establishment of institutionalized mechanisms for cross-departmental coordination. Provide policymakers with ideas for institutional design that can serve as a reference. Second, the operational solutions presented in this study—such as the three-tier administrative MDT problem-solving pathway, performance evaluation and supervision mechanisms, and multi-skilled position training systems—provide policymakers with ideas for institutional design that can serve as a reference, helping to translate macro-level policy directions into actionable implementation guidelines. Finally, the study emphasizes the reform logic of parallel technological empowerment and institutional restructuring, advising policymakers that when promoting hospital informatization, they must simultaneously advance the unification of data standards, the standardization of business processes, and the establishment of cross-departmental governance mechanisms to avoid the pitfall of “digitization” replacing “reengineering.”

However, this study still has certain limitations. First, while research based on a single hospital case study can provide an in-depth analysis of the reform process, the generalizability of its conclusions requires validation through additional case studies. When other hospitals consider adopting this approach, they should evaluate and adapt it in light of their own specific circumstances. Second, the data on reform effectiveness primarily covers the period from 2021 to 2025, necessitating further tracking to assess long-term outcomes and sustainability. Furthermore, this study's assessment of the reform's effectiveness relies entirely on descriptive data from publicly available literature and hospital reports; it did not conduct statistical significance tests, did not include a control group, and did not account for confounding factors that may have influenced hospital operational performance during the same period, such as changes in staffing levels, adjustments to fiscal allocations, and reforms in health insurance reimbursement. This study should interpret the reported changes and reform measures as coinciding in time rather than having a causal relationship. Third, the study did not thoroughly quantify the impact of factors such as implementation costs, personnel resistance, and the external institutional environment. Future research may concentrate on multi-case comparative studies, the quantification of reform costs and benefits, comprehensive analyses of digital tools, and the exploration of the dynamic interplay between reforms and external institutional contexts. In terms of comparative case studies, a stratified sampling approach can be adopted by selecting public hospitals of varying sizes, regions, and types to construct a systematic analytical framework. This will enable the identification of distinctive practices in administrative reform across different hospital categories, the development of universally applicable reform guidelines, and the clarification of key reform priorities and suitable conditions for each hospital type. Regarding cost-benefit analysis, efforts can be made to develop specialized analytical models to systematically calculate the direct and indirect costs of reform, as well as explicit and implicit benefits, thereby establishing a comprehensive evaluation system to provide a reference for reasonably determining the scale of funding allocation. With regard to the analysis of digital tools, it is recommended to employ standardized research methods to conduct an in-depth analysis of the specific mechanisms through which various digital tools function, quantify their actual impact on administrative efficiency, and develop practical application guidelines based on these findings. Regarding the interaction between policy and the environment, it is necessary to continuously monitor policy changes—such as healthcare reform and medical insurance payment reforms—establish a model of interaction between policy and reform, clarify the patterns of alignment between the two, and formulate dynamic adjustment plans accordingly. This will enhance the synergy between reform efforts and the policy environment, thereby strengthening the effectiveness of the reforms.

In the future, China's public hospitals must further break free from traditional management mindsets. Adopting a problem-oriented approach driven by demand, they should pursue systematic administrative process reforms through tiered implementation, deepening digital empowerment, cultivating administrative talent, establishing long-term mechanisms, and implementing scientific assessment systems. Hospitals should categorize themselves by level, focusing on refined, streamlined, and user-friendly reforms during tiered reform implementation. We will strive to shorten approval times and gradually achieve full coverage of mobile services for high-frequency matters, while ensuring that a high percentage of services can be handled through a single service window. For digital empowerment, a unified data middle platform will be established, implementing AI and large language models to facilitate cross-institutional data sharing, which will enhance the efficiency of service delivery and improve patient outcomes by allowing for better coordination of care across different healthcare providers. Aim to reduce the rate of duplicate data entry, increase the level of process automation, and shorten the processing time for cross-agency matters. Regarding administrative talent development, reforms will be incorporated into performance evaluations with dedicated funding. Quarterly assessments and continuous process improvements will be conducted. To establish a sound long-term mechanism, we can improve the quantitative evaluation system, introduce third-party assessments, and link the results to performance evaluations, thereby providing a solid institutional framework and management support for the high-quality development of public hospitals.
